# Identification of Multiple Faults in Gearbox Based on Multipoint Optional Minimum Entropy Deconvolution Adjusted and Permutation Entropy

**DOI:** 10.3390/e20110850

**Published:** 2018-11-06

**Authors:** Huer Sun, Chao Wu, Xiaohua Liang, Qunfeng Zeng

**Affiliations:** 1 The School of Mechanical Engineering, North University of China, Xueyuan Road, Taiyuan 030051, China; 2 Key Laboratory of Education Ministry for Modern Design and Rotor-Bearing System, Xi’an Jiaotong University, Xi’an 710049, China

**Keywords:** multipoint optional minimum entropy deconvolution adjusted, permutation entropy, gearbox, fault detection

## Abstract

The weak compound fault feature is difficult to extract from a gearbox because the signal components are complex and inter-modulated. An approach (that is abbreviated as MRPE-MOMEDA) for extracting the weak fault features of a transmission based on a multipoint optimal minimum entropy deconvolution adjustment (MOMEDA) and the permutation entropy was proposed to solve this problem in the present paper. The complexity of the periodic impact signal was low and the permutation entropy was relatively small. Moreover, the amplitude of the impact was relatively large. Based on these advantages, the multipoint reciprocal permutation entropy (MRPE) was proposed to track the impact fault source of the weak fault feature in gearbox compound faults. The impact fault period was indicated through MRPE. MOMEDA achieved signal denoising. The optimal filter coefficients were solved using MOMEDA. It exhibits an outstanding performance for noise suppression of gearbox signals with a periodic impact. The results from the transmission show that the proposed method can identify multiple faults simultaneously on a driving gear in the 4th gear of the transmission.

## 1. Introduction

A gearbox plays an important role in the transmission system, such as in the automobile industry. Moreover, the manufacturing cost of gearboxes is relatively high, so early fault detection is important. The structure of a gearbox is complex and high assembly standards are required. A multi-fault diagnosis has been mostly concentrated on gearbox faults such as gear and rolling bearings [1,2]. However, the simultaneous detection of multiple faults is still a big challenge in the monitoring and diagnosis of gearbox faults. When there are multiple faults in the transmission, the severity and characteristics of each fault are often different and the characteristics of weak faults are easily missed. Therefore, an indicator to show the existence of the fault is necessary. Many gearbox fault types are impulse-like, therefore, the indicator of the impact failure in a gearbox was studied in this paper.

The gearbox includes many parts, such as gears, bearings, shafts and other parts. The vibration signals acquired by sensors are derived from the gears, bearings and other parts. It is difficult to identify the source of a fault [3]. In the early fault phase of the gearbox, the impact of gear and bearing faults is submerged in intensive background noise. Moreover, the gear impact fault and bearing fault signals show a similar impact modulation phenomenon in the time domain [4]. The detection of gear faults is usually limited to identify the stage that contains the faulty gear, not the faulty gear itself [5,6]. The local defects on a gear tooth may change the contact stiffness, the bending stiffness as well as the shear stiffness, which produces a series of impulses in the vibration signal during meshing. A wavelet transformation selects the frequency band with the maximum kurtosis to filter. However, it takes a frequency range of the signal and discards the rest of the information [7]. The measured vibration signals are considered as a convolution process of the malfunction inducing periodic impact signals and a resonant response of the mechanical components. Therefore, deconvolution is an effective way to restore the impulses. The minimum entropy deconvolution (MED) method has been shown to be an effective deconvolution method and has been employed in rotating machinery fault diagnosis [8]. However, it still has some limitations [9,10]. A deconvolution method called maximum correlated kurtosis deconvolution (MCKD) was proposed in 2012 to address some of the limitations of MED [11]. Although it can deconvolve periodic impulses, there was still an iterative solution MCKD [12]. A method called the multipoint optional minimum entropy deconvolution adjustment (MOMEDA) was further developed from the minimum entropy deconvolution (MED) method. It has been proven as an efficient tool to extract the periodic impulses in the diagnosis of gearbox faults [13]. The deconvolution method can restore a specific input of the system under the unknown conditions of the system but the characteristics of the periodic impact are not extracted. Therefore, it is necessary to calculate the corresponding filter coefficients with a specific index. MED was proposed using kurtosis; MCKD was proposed using correlated kurtosis (CK); MOMEDA was proposed using MKurt as the fault indicator. However, these methods are based on kurtosis, ignoring the intrinsic characteristics of the signals.

Besides the feature extraction from the perspective of fault feature frequency, other technologies are being applied in the field of fault diagnosis. From the perspective of the probability theory, the realization of fault recognition based on the entropy of different fault states has also become a new research direction in the field of fault diagnosis. Based on the information theory, it is well-known that the complexity of a system can be estimated by means of entropy computation since the entropy represents the complexity estimation of the system measurements. There are different types of entropy definitions, such as the approximate entropy [14], sample entropy [15], spectral entropy [16], pattern spectrum entropy [17], and permutation entropy [18,19], which have been utilized to evaluate the regularity or disorderliness of mechanical and physiological systems. Therefore, the statistical quantification approach has been employed to diagnose machinery faults.

Permutation entropy (PE) was used to measure the randomicity and to detect dynamical changes of a time series [20]. As one of the useful concepts, PE can reflect the dynamic behavior of a nonlinear and nonstationary time series [21]. Compared with the SampEn, its performance was improved with the advantage of fewer parameters and an increased computational efficiency [22]. In addition, it is small for impulse-like signals. In the proposed method, the PE of the vibration signal was calculated to detect the impact malfunction periods of a gearbox.

The present paper is organized as follows. Section 2 reviews the basic process of MKurt-MOMEDA. In Section 3, MRPE-MOMEDA is proposed for the diagnosis of multiple faults. The multipoint reciprocal permutation entropy was calculated as the index of an impulse series. Section 4 presents the procedure and preliminary validation of MRPE-MOMEDA using simulated gearbox vibration data containing compound-faults. Additionally, a performance analysis was executed. Then, in Section 5, real datasets from the auto transmission further validate the effectiveness of the proposed method by comparing it with the origin MKurt-MOMEDA. The conclusions are summarized in Section 6.

## 2. The Multipoint Optimal Minimum Entropy Deconvolution Adjustment

The response signal was collected as in Equation (1):

(1) y(n)=h(n)∗x(n)+e(n)

where e(n) is the noise, x(n) is the impact sequence, h(n) is the transfer function and y(n) is the collected vibration signal.

The MOMEDA algorithm was used to find an FIR filter that recovered the input signal x(n) by the output signal y(n).

The deconvolution of multi-pulse targets at known locations and the recognition of continuous shock pulses was improved by MOMEDA.

(2) $$\mathrm{Multi}\;\text{D-Norm}=\mathrm{MDN}(y,t)=\frac{1\times t^{T}y}{\left\|t\right\|\left\|y\right\|}$$

MOMEDA is the maximization problem for solving the maximum of the multi-D norm.

(3) $$\underset{\vec{f}}{\max}\mathrm{MDN}(y,t)=\underset{\vec{f}}{\max}\frac{t^{T}y}{\left\|y\right\|}$$

where the target $\vec{t}$ is a constant vector that defines the targets pulse position and weights. McDonald proved that the direct calculation method of the filter can be obtained [13].

Since $\vec{y}=X_{0}^{T}\vec{t}$ and assuming ${\left(X_{0}X_{0}^{T}\right)}^{-1}$ exists:

(4) $$\frac{{\vec{t}}^{T}\vec{y}}{{\left\|\vec{y}\right\|}^{2}}\vec{f}={\left(X_{0}X_{0}^{T}\right)}^{-1}X_{0}\vec{t}.$$

Since multiple values of f are also solutions to Equation (4), multiple values of $\vec{f}={\left(X_{0}X_{0}^{T}\right)}^{-1}X_{0}\vec{t}$ are solutions to the MOMEDA problem.

(5) $$\vec{f}={\left(X_{0}X_{0}^{T}\right)}^{-1}X_{0}\vec{t}$$

$$X_{0}={\left[\begin{array}{ccccc} X_{L} & X_{L+1} & X_{L+2} & \cdots & X_{N}\\ X_{L-1} & X_{L} & X_{L+1} & \cdots & X_{N-1}\\ X_{L-2} & X_{L-1} & X_{L} & \cdots & X_{N-2}\\ \vdots & \vdots & \vdots & \ddots & \vdots \\ x_{1} & x_{2} & x_{3} & \cdots & x_{N-L+1} \end{array}\right]}_{L\times N-L+1}$$.

The output

(6) $$\vec{y}=X_{0}^{T}\vec{f}.$$

Multipoint kurtosis (MKurt) was introduced as a measure of the feature extraction for a multi-stage transmission gearbox with wide frequency distributions and a multi-fault period:

(7) $$\mathrm{MKurt}=\frac{{\left(\sum_{n=1}^{N-L}t_{n}^{2}\right)}^{2}{\sum_{n=1}^{N-L}\left(t_{n}y_{n}\right)}^{4}}{\sum_{n=1}^{N-L}t_{n}^{8}{\left(\sum_{n=1}^{N-L}y_{n}^{2}\right)}^{2}}.$$

The procedure for the detection of gearbox faults using MKurt-MOMEDA is summarized. The algorithmic framework of MOMEDA for the detection of gearbox faults is listed as follows [13]:

- Step 1: Loading the raw vibration signal, x, measured by an accelerometer and the range of the fault period, T.
- Step 2: Selecting the appropriate filter length, L, and window size.
- Step 3: Calculating $X_{0}$,$X_{0}^{T}$ and ${\left(X_{0}X_{0}^{T}\right)}^{-1}$ from the signal, x. Yielding the optimal filter from Equation (3). Obtaining the filtered signal, y, from Equation (6).
- Step 4: Building an array of target impulse train vectors, separated by the periods, t.
- Step 5: Applying the window function to the target vectors.
- Step 6: Calculating the spectrum of outputs.
- Step 7: Calculating the spectrum of MKurt values for each output.
- Step 8: Finding the maximum value of MKurt and the best match output.
- Step 9: Enveloping the spectrum analysis.

## 3. The Proposed Method for The Gearbox Fault Diagnosis

### 3.1. Basic Principle of Permutation Entropy

As a measure of the complexity and randomness of a one-dimensional time series, permutation entropy was used to detect the kinetic mutation of the system [20]. Setting a time sequence as {x(i),i=1,2,3,⋯,n}, according to the TAKENS phase space reconstruction method, the matrix was reconstructed. 

For any arbitrary time series {x(i),i=1,2,3,⋯,n}, according to the embedding theorem, the matrix was defined as:

(8) $$\left[\begin{array}{cccc} x(1) & x(1+\tau ) & \cdots & x(1+(m-1)\tau )\\ x(1) & x(2+\tau ) & \cdots & x(2+(m-1)\tau )\\ \vdots & \vdots & \ddots & \vdots \\ x(K) & x(K+\tau ) & \cdots & x(K+(m-1)\tau ) \end{array}\right],\;j=1,2,3,\cdots ,K.$$

Among them, m and τ are the embed dimension and the delay time, respectively. There are K reconstructed components, K=n−(m−1)τ. Each row in the matrix was viewed as a reconstructed component, with a total of reconstructed components. The elements in each reconstructed component were arranged in ascending order according to their numerical value. In addition, the index of each column in the reconstructed component before sorting was extracted to form a sequence of symbols. There are m! kinds of different symbol sequences in m-dimensional phase space mapping used to calculate the probability that the symbol sequence of the k sort of an arrangement appears, denoted by $P_{k}$. Then, the permutation entropy of the time series can be obtained using the following formula:

(9) $$H_{p}=-\sum_{i=1}^{k}P_{i}\ln P_{i}.$$

When $P_{i}=1/m!$, $H_{p}$ reaches its maximum value ln(m!). For convenience, $H_{p}(m)$ is usually normalized with ln(m!), that is

(10) $$0\leq H_{p}=H_{p}/\ln(m!)\leq 1.$$

The size of $H_{p}$ reflects the complexity and randomness of the time series [22]. The randomness of the vibration signal and the permutation entropy of the mechanical equipment becomes high under good conditions. However, when mechanical equipment has a fault, the randomness of the vibration signal reduced and the complexity became smaller. Therefore, the permutation entropy can indicate whether there are impulse components in the signal. The amplitude of the pulse signal was relatively large. The signal amplitude was considered in the permutation entropy.

### 3.2. Multipoint Reciprocal Permutation Entropy

(11) $$\mathrm{MPE}=\frac{H_{p}}{\sum_{n=1}^{N-L}y_{n}^{2}\sum_{n=1}^{N-L}\left|t_{n}\right|}$$

The present work was inspired by MKurt. In the proposed method, the MKurt index of the MOMEDA was replaced by the multipoint permutation entropy (MRPE). It was defined on the basis of the permutation entropy. In addition, the consideration of the amplitude of a time series and the location of the pulse in the signal was revealed. For a more intuitive display the reciprocal of MPE, that is MRPE, was calculated:

(12) $$\mathrm{MRPE}=\frac{1}{MPE}.$$

**Figure 1:**
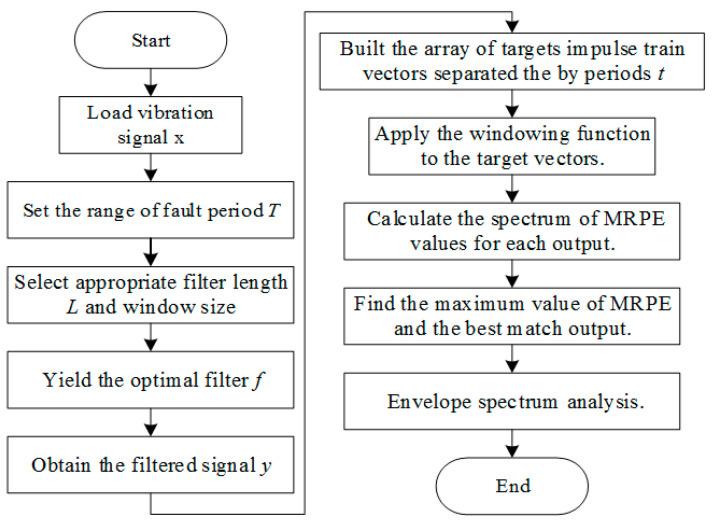
Flow of MRPE-MOMEDA.

The MRPE-MOMEDA fault diagnosis process is shown in Figure 1.

## 4. Simulations

Simulated gear meshing signals are listed:

(13) $$h(t)=\sum_{m=0}^{M}X_{m}(1+a_{m}(t))\cos(2\pi mzf_{r}t+\phi_{m}+b_{m}(t))$$

where M is the harmonic component number of a meshing gear. $f_{r}$ is the rotation frequency. z is the gear teeth, $X_{m}$ and $\phi_{m}$ are the magnitude and phase of the engaged harmonic, respectively. $a_{m}$ and $b_{m}$ are the modulation functions of the first harmonic amplitude and phase, respectively. When the shaft rotates one revolution, the teeth with local faults mesh once. $a_{m}$ and $b_{m}$ are periodic functions whose frequency is the rotation frequency and its harmonics. Therefore, $a_{m}$ and $b_{m}$ contain the gear fault information.

The simulated rolling bearing fault signals are shown, as follows, in Equation (14):

(14) $$s(t)=\sum_{m=0}^{M}B_{m}\exp[-\beta (t-mT_{p})]\times \cos[2\pi f_{re}\times (t-mT_{p})]u(t-mT_{p})$$

where M is the number of shocks in the periodic impact signal, $B_{m}$ is the amplitude of the *m* impact, and β is the decay rate of the impact. In order to simulate the impact interval, the fault characteristic frequency $f_{c}$ was satisfied with $f_{c}=1/T_{p}$. $f_{re}$ is the resonance frequency. $u(t-mT_{p})$ is a unit step function.

The vibration of a normal gearbox is mainly caused by mesh force and the vibration signal x(t) is dominated by the tooth pair mesh frequency and its harmonics.

In summary, the vibration signals of the meshing component and the periodic impact components are a mixed signal when both the bearing and the gear are have failed locally in the gearbox.

In order to verify the advantages of MRPE-MOMEDA to extract pulses by comparing with MKurt-MOMEDA, the following simulation signal was used for the experimental analysis. The simulation signal was:

(15) $$x(t)=h(t)+s_{1}(t)+s_{2}(t)+n(t).$$

**Figure 2:**
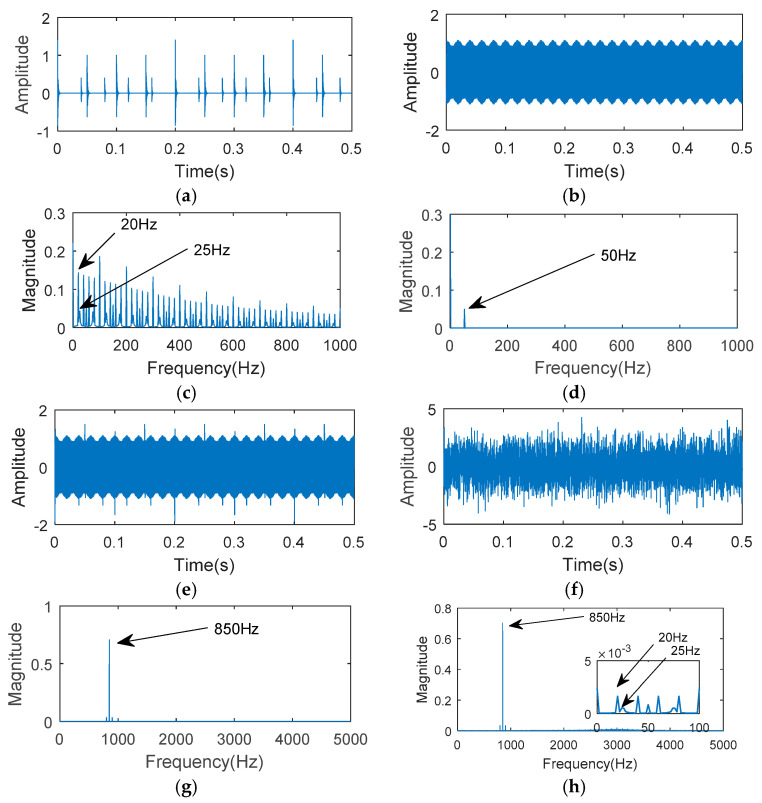
Simulated vibration signals and spectra: (**a**) Simulated impact signal; (**b**) simulated gear meshing signal; (**c**) the impact signal envelope spectrum [23]; (**d**) the gear meshing signal envelope spectrum; (**e**) the simulated hybrid signal; (**f**) the simulated vibration signal with 0 dB noise; (**g**) the simulated hybrid signal FFT; (**h**) the simulated hybrid signal with 5 dB noise FFT.

The simulation signal x(t) and its spectrum are given in Figure 2 (sampling length N=5000). A white Gaussian noise function n(t) was added to the simulation signal x(t), the impact component was completely merged and barely recognizable. Setting $f_{r}=50\;\mathrm{Hz}$, $f_{re}=3000\;\mathrm{Hz}$, $T_{1}=0.05$, $y_{1}=1$, and $T_{2}=0.04$, $y_{2}=0.4$. As the amplitude of $s_{2}(t)$ was much smaller than that of $s_{1}(t)$, in this simulation, it was difficult to distinguish the impact component $s_{2}(t)$.

**Figure 3:**
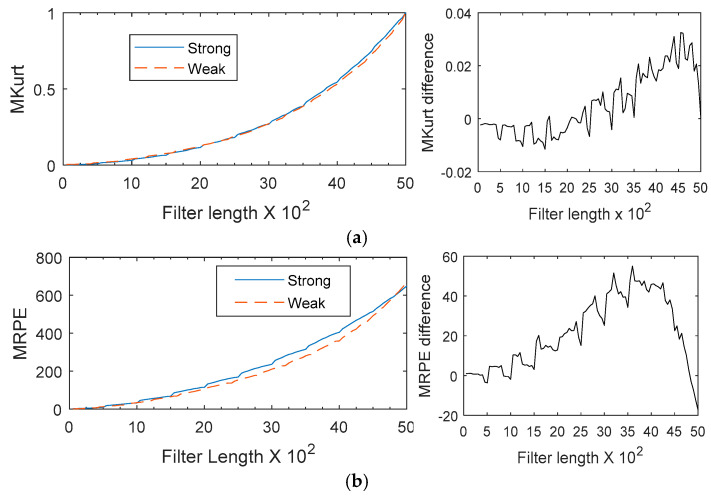
MOMEDA fault detection results versus filter length *L*: (**a**) MKurt; (**b**) MRPE.

The simulation signal was filtered using the MKurt-MOMEDA and MRPE-MOMEDA methods respectively. Figure 3a illustrates the relationship among the filter length *L*, the fault metric MKurt, and the MKurt difference between the strong faulty and weak faulty states under each setup condition. Figure 3b illustrates the relationship among the filter length *L*, the fault metric MRPE, and the MRPE differences between the strong faulty and weak faulty states under each setup condition. Under all filter lengths and machine conditions, the MKurt value and the MRPE value under strong faulty states were larger than the values of the weak faulty state. A filter length of *L* = 500 was chosen for the results as a balance between the lower variance associated with smaller filter lengths and a higher MKurt difference associated with larger filter lengths.

**Figure 4:**
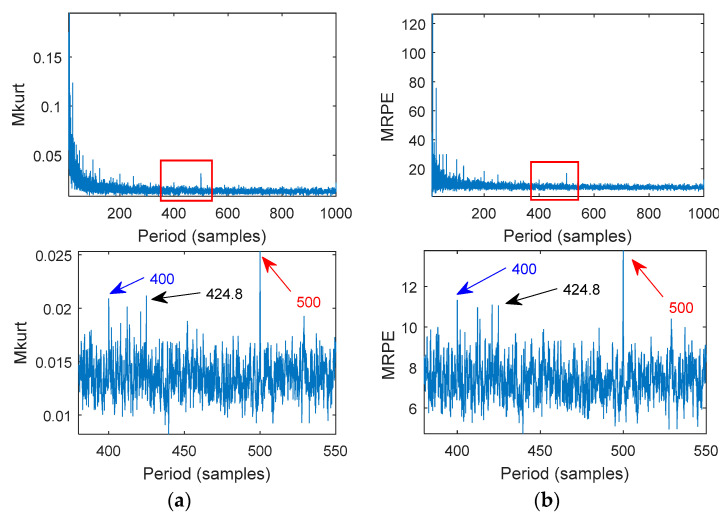
MOMEDA compared to MKurt with MRPE: (**a**) An MKurt-MOMEDA spectrum; (**b**) an MRPE-MOMEDA spectrum.

**Figure 5:**
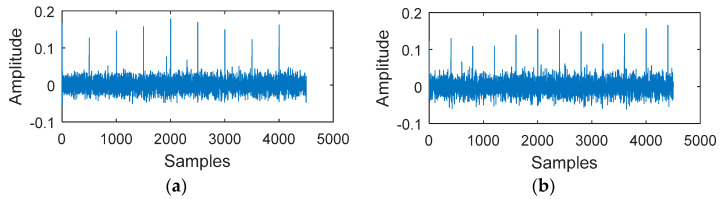
The MOMEDA filtered Signal: (**a**) The MOMEDA filtered signal (Ts = 500); (**b**) the MOMEDA filtered signal (Ts = 400).

MOMEDA was used to calculate several consecutive target vectors and further distinguish between the fault periods and non-fault periods of its surroundings. Figure 4 shows the MKurt and MRPE values of the simulated signal. In general, both methods can indicate the strong fault impact period (500). However, MRPE after local amplification showed that the MRPE value at the weak fault impact period (400) was greater than the MRPE value of the adjacent noise. Therefore, the weak fault impact period (400) was more pronounced. Figure 5 plots the filtered signal from MOMEDA. Figure 5a shows the strong impact signal extracted from the original signal with an interval sampling number of 500. Figure 5b shows a weak impact signal extracted from the original signal with an interval sampling number of 400.

## 5. Experimental Analysis

**Figure 6:**
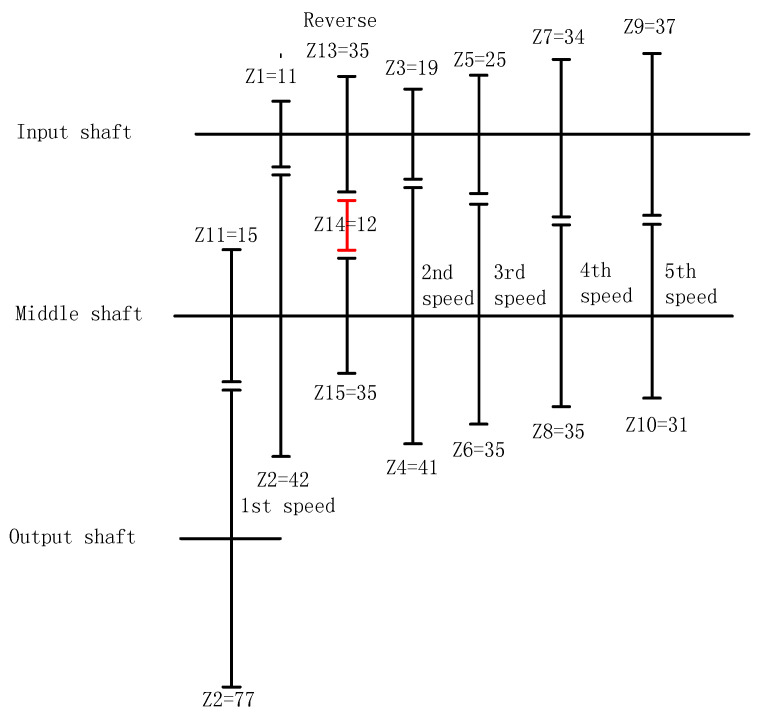
Drive structural diagram and number of gear teeth in each gear.

There were auto transmissions in the present work. The gearbox had five forward gears and one inverted gear. Figure 6 shows the corresponding relationship between the gears.

The vibration signals acquired by the acceleration sensor contained all parts of the transmission, such as the meshing gear and rolling bearings. The distances between the acceleration sensor and each shaft were different according to the internal structure of the transmission. The vibration signals acquired from the shafts had different vibration energy levels.

**Figure 7:**
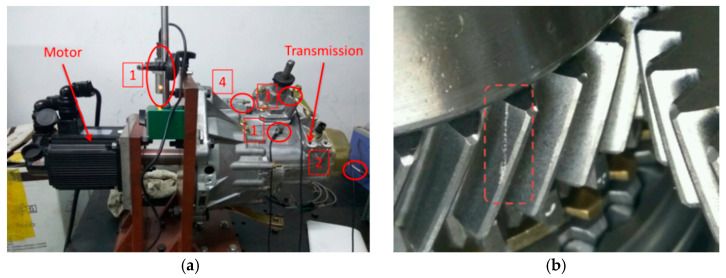
The test bench and the tested transmission: (**a**) The five-speed transmission and (**b**) the gear local fault.

The experiments were conducted on a five-speed transmission. The structural diagram of the transmission is shown in Figure 7a. A three-phase AC servo motor output shaft, a transmission input shaft and a drive transmission operation were coupled. The vibration signals were acquired using an NI Compact DAQ 9234. The input shaft speed was 2800 r/min and the transmission was shifted to the fourth gear. The operating parameters are shown in Table 1. Figure 7b shows the location and size of the fourth gear failures.

For the MOMEDA feature extraction, the pre-calculated model was built on the basis of the faulty frequencies for the different mechanical parts. Table 1 shows the operating frequencies regarding the rotational speed of the motor for the different mechanical parts. Table 2 shows the transmission bearing fault feature template and the bearing parameters.

**Figure 8:**
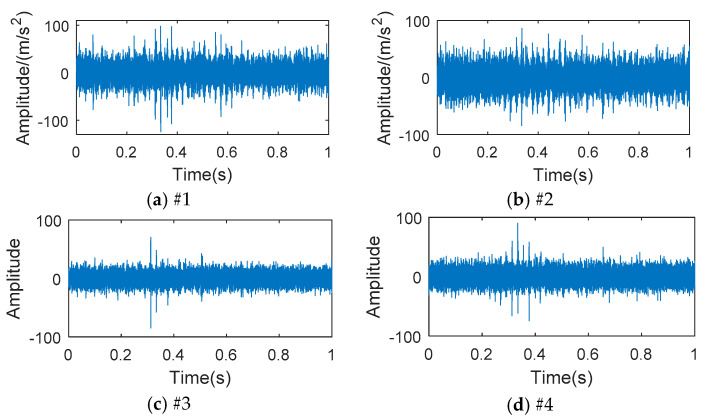
The vibration signals acquired at each measurement point from the transmission in 4th gear: (**a**) the first measurement point; (**b**) the second measurement point; (**c**) the third measurement point; (**d**) the fourth measurement point.

Four acceleration sensors (PCB352C33) were attached to the transmission box, picking up vibration signals on the surface of the transmission. Figure 7a shows the position of each acceleration sensor. The sampling frequency was 25.6 k/s and there were 51,200 sampling points. Figure 8 shows the vibration signal acquired for each measurement point. The estimation period of the vibration signal is shown in Table 3.

**Figure 9:**
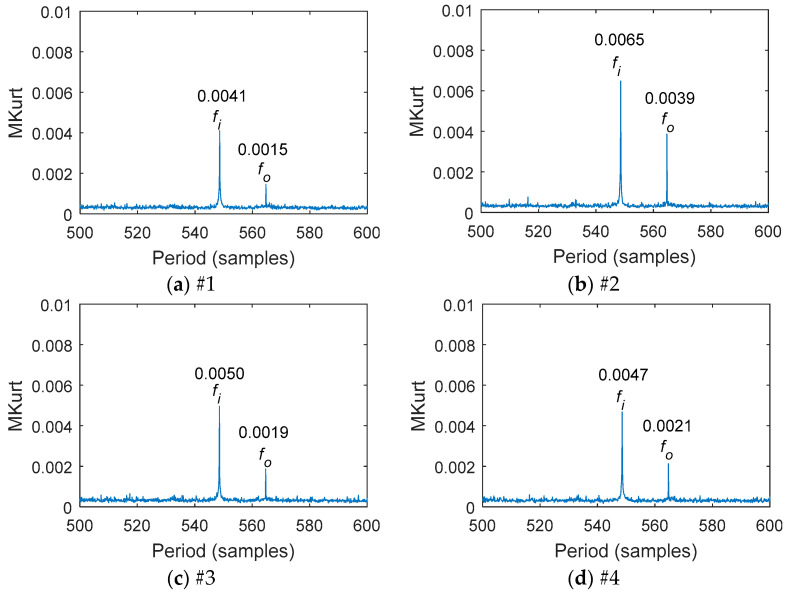
MKurt of the period range [500,600] at each measurement point: (**a**) the first measurement point; (**b**) the second measurement point; (**c**) the third measurement point; (**d**) the fourth measurement point.

**Figure 10:**
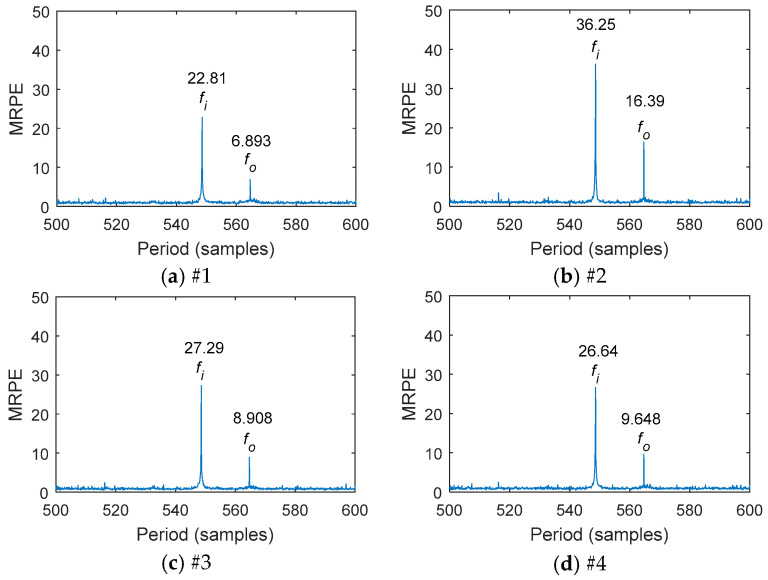
MRPE of the period range [500,600] at each measurement point: (**a**) the first measurement point; (**b**) the second measurement point; (**c**) the third measurement point; (**d**) the fourth measurement point.

As shown in Figure 9 and Figure 10, the multipoint kurtosis values and the multipoint reciprocal permutation entropy values of each vibration measurement point signal were calculated. It was found that the multipoint kurtosis of the input shaft was greater than that of the output shaft. The multipoint kurtosis value and multipoint reciprocal permutation entropy value of the second measurement point were greater than those of the other measurement points. The MRPE values of different points were different. The difference of the MKurt value among different points was small. The transmission forward gear was a helical gear and the axial vibration was greater than the radial vibration. Therefore, the axial impact was larger than the radial impact when the tooth surface was damaged. Based on the above results, the signal of the second measurement point for feature extraction was selected. 

**Figure 11:**
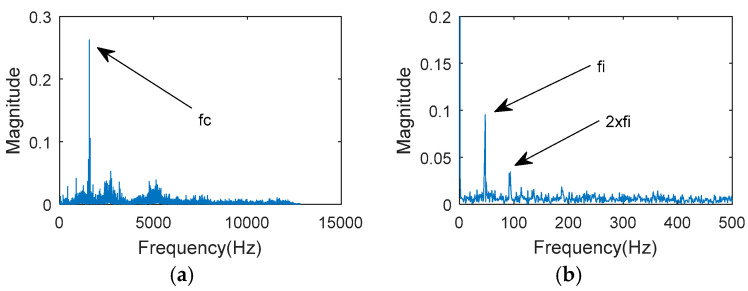
Vibration signal acquired at the 2nd measurement point:(**a**) The FFT spectrum; (**b**) the Hilbert envelope spectrum.

The spectral analysis and Hilbert envelope analysis results are shown in Figure 11a,b. The gear meshing frequency was found but there was no bearing fault frequency. The following MOMEDA method was used for noise reduction. When the filter length L=600, MOMEDA filtering was carried out for the 2nd measurement point.

**Figure 12:**
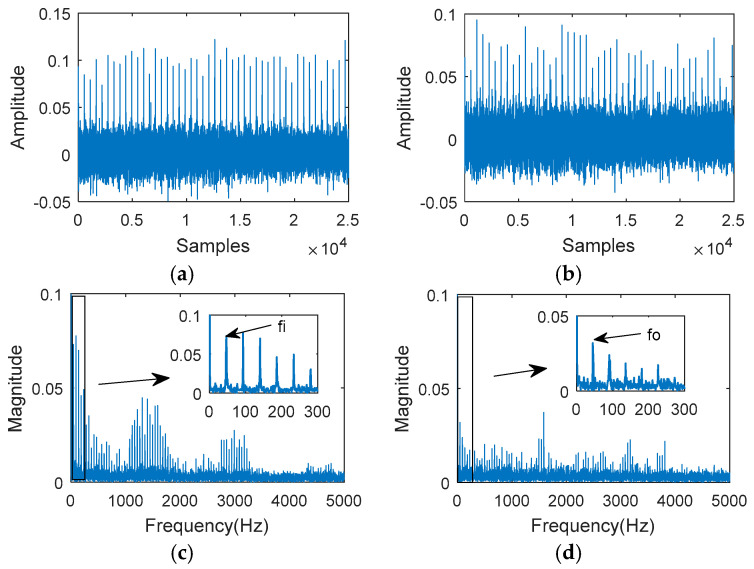
The MOMEDA filtered signal analysis: (**a**) The filtered signal (Ts = 548.6); (**b**) the filtered signal (Ts = 564.7); (**c**) the envelope spectrum; (**d**) the envelope spectrum.

The MOMEDA filtered signal is shown in Figure 12a,b. The envelope spectrum analysis of the input shaft impact filtered signal and the output shaft impact filtered signal are shown in Figure 12c,d. Figure 12c,d shows clearly the rotation frequencies of the input shaft and the output shaft. The analysis results show that the impact failure period indicated by MRPE is reasonable.

According to the analysis, compared to the driven gear faults in the 4th gear of the transmission, the drive shaft faults were more serious. The analytical results show that MRPE-MOMEDA was beneficial for extracting weak impact signals and MRPE was helpful to trace multi-faults. It was concluded that the proposed method accurately identified the weak impulsive signal caused by the gear damage.

## 6. Conclusions

The compound fault diagnoses of a gearbox were investigated during normal operation. The weak fault feature extraction method based on MOMEDA and permutation entropy was studied in the present paper. The MOMEDA algorithm with MRPE was used to pre-process the vibration signals to separate the weak fault signals in the compound faults. The results of the present work can be summarized as follows:

1. The diagnoses of weak impact faults in complex faults were achieved using MOMEDA combined with a spectrum analysis.
2. The MKurt-MOMEDA and MRPE-MOMEDA were able to identify the multi-faults of rotating machinery using simulation and experimental analysis.
3. A comparison of the fault indication between MKurt-MOMEDA and MRPE-MOMEDA was investigated. Compared to MKurt, MRPE had an excellent tracking ability of the sources of impact faults under the same fault conditions. The MRPE values of the different points were greater. The difference of the MKurt values among the different points was small.
4. The impact fault frequencies of 46.7 Hz and 45.3 Hz were extracted from the transmission vibration signal by MRPE-MOMEDA.
5. Weak feature extraction in composite faults was difficult. There were fewer test failure samples and more different types of faults need to be further verified in the future.

## Figures and Tables

**Table 1:** The operation parameters the transmission in 4th gear.

Parameter	The 4th Gear Pair		Constantly Meshed Gear Pair	
	Drive Wheel	Driven Wheel	Drive Wheel	Driven Wheel
Gear number	34	35	15	77
Rotational frequency (Hz)	46.7	45.3	45.3	8.8
Mesh frequency (Hz)	1586.7		679.5	

**Table 2:** Defect frequencies of the rolling bearing of the transmission.

Position	f_oc_	f_bc_	f_c_	f_ic_
Input shaft	2.504	1.616	0.358	4.494
	3.454	2.036	0.384	5.544
Middle shaft	7.32	3.324	0.3792	11.0808
	6.61	2.9742	0.3792	9.914
Output shaft	0.891	0.576	0.081	1.2398
	0.6748	0.435	0.075	1.0282

**Table 3:** Theoretical estimation periods.

Data	Speed (r/min)	Sample Rate (k/s)	Frequency/Hz	Samples
1	2800	25.6	46.7	548.2
2	2800	25.6	45.4	563.8

## Abbreviations

The following abbreviations are used in this manuscript:

MOMEDA	Multipoint Optional Minimum Entropy Deconvolution Adjusted
MKurt	Multipoint Kurtosis
MPE	Multipoint Permutation Entropy
MRPE	Multipoint Reciprocal Permutation Entropy
SampEn	Sample Entropy
FIR	Finite Impulse Response
FFT	Fast Fourier Transformation
